# Classifying Schizophrenia Cases by Artificial Neural Network Using Japanese Web-Based Survey Data: Case-Control Study

**DOI:** 10.2196/50193

**Published:** 2023-11-15

**Authors:** Yupeng He, Masaaki Matsunaga, Yuanying Li, Taro Kishi, Shinichi Tanihara, Nakao Iwata, Takahiro Tabuchi, Atsuhiko Ota

**Affiliations:** 1 Department of Public Health Fujita Health University School of Medicine Toyoake Japan; 2 Department of Public Health and Health Systems Nagoya University Graduate School of Medicine Nagoya Japan; 3 Department of Psychiatry Fujita Health University School of Medicine Toyoake Japan; 4 Department of Public Health Kurume University School of Medicine Kurume Japan; 5 Cancer Control Center Osaka International Cancer Institute Osaka Japan

**Keywords:** artificial neural network, schizophrenia, prevalence, Japan, web-based survey, mental health, psychosis, machine learning, epidemiology

## Abstract

**Background:**

In Japan, challenges were reported in accurately estimating the prevalence of schizophrenia among the general population. Retrieving previous studies, we investigated that patients with schizophrenia were more likely to experience poor subjective well-being and various physical, psychiatric, and social comorbidities. These factors might have great potential for precisely classifying schizophrenia cases in order to estimate the prevalence. Machine learning has shown a positive impact on many fields, including epidemiology, due to its high-precision modeling capability. It has been applied in research on mental disorders. However, few studies have applied machine learning technology to the precise classification of schizophrenia cases by variables of demographic and health-related backgrounds, especially using large-scale web-based surveys.

**Objective:**

The aim of the study is to construct an artificial neural network (ANN) model that can accurately classify schizophrenia cases from large-scale Japanese web-based survey data and to verify the generalizability of the model.

**Methods:**

Data were obtained from a large Japanese internet research pooled panel (Rakuten Insight, Inc) in 2021. A total of 223 individuals, aged 20-75 years, having schizophrenia, and 1776 healthy controls were included. Answers to the questions in a web-based survey were formatted as 1 response variable (self-report diagnosed with schizophrenia) and multiple feature variables (demographic, health-related backgrounds, physical comorbidities, psychiatric comorbidities, and social comorbidities). An ANN was applied to construct a model for classifying schizophrenia cases. Logistic regression (LR) was used as a reference. The performances of the models and algorithms were then compared.

**Results:**

The model trained by the ANN performed better than LR in terms of area under the receiver operating characteristic curve (0.86 vs 0.78), accuracy (0.93 vs 0.91), and specificity (0.96 vs 0.94), while the model trained by LR showed better sensitivity (0.63 vs 0.56). Comparing the performances of the ANN and LR, the ANN was better in terms of area under the receiver operating characteristic curve (bootstrapping: 0.847 vs 0.773 and cross-validation: 0.81 vs 0.72), while LR performed better in terms of accuracy (0.894 vs 0.856). Sleep medication use, age, household income, and employment type were the top 4 variables in terms of importance.

**Conclusions:**

This study constructed an ANN model to classify schizophrenia cases using web-based survey data. Our model showed a high internal validity. The findings are expected to provide evidence for estimating the prevalence of schizophrenia in the Japanese population and informing future epidemiological studies.

## Introduction

Schizophrenia is a common mental illness that disrupts a person’s thinking processes, perceptions, emotional responsiveness, and social interactions [1]. Estimates of international prevalence range from 0.33% to 0.75% [2,3]. The lifetime prevalence and median 12-month prevalence of schizophrenia were reported to be 0.33% and 0.48%, respectively [4]. In Japan, the point prevalence of schizophrenia, including schizotypal and delusional disorders, is approximately 0.7% according to national data from a patient survey [5]. While the real prevalence was considered quite different owing to the obstacles when operating the investigation. Patients with mild cases might not seek medical attention, and some cases are diagnosed as schizophrenia just for prescriptions to pass the medical insurance review.

We envisioned whether the prevalence of schizophrenia in individuals could be predicted by several factors and estimated the prevalence in the general population. By retrieving data from previous systematic reviews and meta-analyses, we confirmed that individuals with schizophrenia experience poor subjective well-being and various physical, psychiatric, and social comorbidities. For instance, studies conducted in Canada and the United States [6,7] have reported that young adults with schizophrenia tend to experience poorer subjective well-being and lower life satisfaction. Additionally, individuals with schizophrenia are prone to higher risk of noncommunicable diseases and experience poor oral health [8-10]. Patients with schizophrenia frequently exhibit symptoms of depression and experience sleep disorders [11,12]. Furthermore, individuals with schizophrenia typically have lower employment rates [11] and exhibit challenges in social cognition [13]. These factors are strongly associated with the incidence and existence of schizophrenia [14].

Machine learning techniques have recently drawn increasing attention in psychiatric studies. Birnbaum et al [15] built machine learning diagnostic and relapse classifiers for schizophrenia based on internet search activity (timing, frequency, and content), which achieved the area under the curve value of 0.74 and 0.71, respectively. Natural language processing has been applied to detect schizophrenia signs from social media content with extremely high accuracy [16]. Lejeune et al [17] concluded in their review that studies using social media to diagnose mental disorders were promising, while limitations included lack of clinical diagnostic data, small sample size, and heterogeneity in study quality. Previous studies have reported the effectiveness of detecting various types of mental disorders [18,19]. In other epidemiological fields, machine learning techniques also manifested promise, especially excelling at dealing with large-scale data [20,21]. We recently researched developing estimation methods for schizophrenia among the Japanese population [22]. Data were collected using a large-scale web-based survey. Individuals who participated in this survey were asked to answer questions about demographics, health-related backgrounds, physical comorbidities, psychiatric comorbidities, and social comorbidities. Compared with classical epidemiological surveys, web-based surveys make it easy to reach a large sample size and amount of data. Few studies have referred to the precise prediction of schizophrenia using large-scale web-based surveys.

If schizophrenia cases could be classified by variables of demographic and health-related backgrounds, it would be possible to estimate schizophrenia cases among general population, who are not seeking psychiatric care (namely, whose psychiatric syndromes are unknown). Therefore, we aimed to construct a machine learning model that can accurately classify the schizophrenia case and verify its generalizability.

## Methods

### Study Design: Participants and Survey Items

A prevalence case-control study was conducted using an internet research agency’s pooled panel (Rakuten Insight, Inc, incorporated approximately 2.3 million panelists by 2022) [23]. Participants’ ages were restricted from 20 to 75 years. Individuals who participated in this study answered a web-based survey.

Among participants who currently have schizophrenia, 5584 individuals who self-reported schizophrenia were sampled in the Rakuten Insight disease panel [24]. A total of 3256 respondents answered the following four questions before the survey: (1) are you currently experiencing schizophrenia only; schizophrenia and migraine; schizophrenia and a sleep disorder; or schizophrenia, migraine, and a sleep disorder? (2) Have you experienced auditory hallucinations lasting more than 1 month? (3) Have you never used stimulants or other illegal drugs and have never been an alcoholic? (4) Have you experienced your first auditory hallucination lasting more than 1 month at less than 60 years of age? Those who answered “yes” to all 4 questions were considered to have schizophrenia. Therefore, 223 participants who currently had schizophrenia were included in the survey.

For participants who do not currently have schizophrenia, all 28,000 participants in the Japan COVID-19 and Society Internet Survey (which was also conducted using the Rakuten Insight Panel) [25] were sampled. A total of 6656 respondents answered the following four questions before the survey: (1) are you currently experiencing mental illness? (2) Have you experienced a mental illness in the past? (3) Have you experienced auditory hallucinations? (4) Have you ever used stimulants or other illegal drugs, been alcoholic, or received psychiatric treatment? Those who answered “no” to all 4 questions were considered to not have schizophrenia. Therefore, 1776 participants who did not currently have schizophrenia were included in the survey.

In the survey, 223 participants with schizophrenia and 1776 healthy controls answered a self-administered questionnaire. The question items were designed to assess (1) demographic and health-related backgrounds and physical comorbidities, (2) psychiatric comorbidities, and (3) social comorbidities. Answers to the question items were formatted to 1 response variable (diagnosed with schizophrenia, “yes” or “no”) and 75 feature variables (demographic, health-related backgrounds, physical comorbidities, psychiatric comorbidities, and social comorbidities). Details of the study participants and variable definitions have been published elsewhere [26] and are described in Multimedia Appendix 1.

### Artificial Neural Network

**Figure 1:**
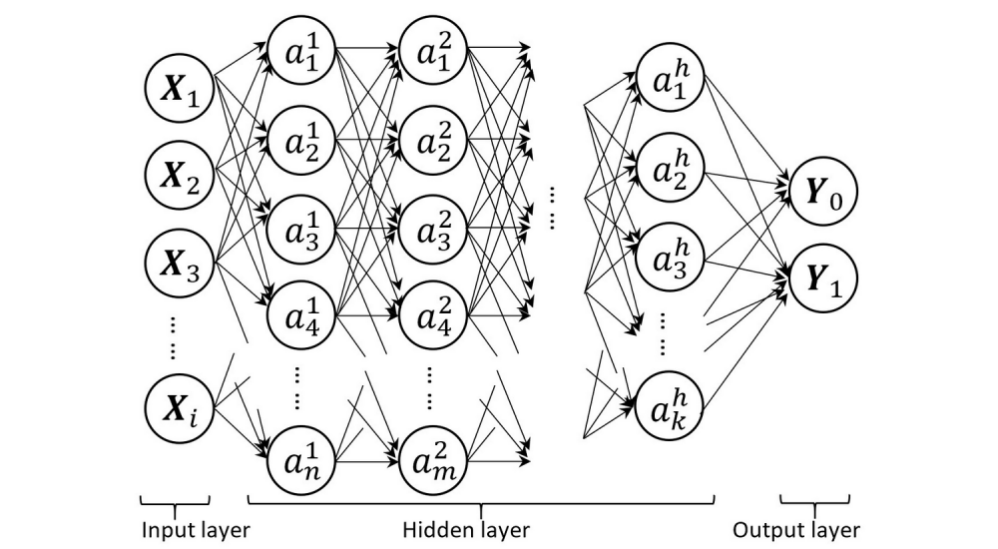
Structure of the artificial neural network. *X* refers to each of the feature variables. *a* refers to neurons in hidden layers. *Y* refers to response variables during training process and prediction results during test process.

An artificial neural network (ANN) is a computing system that imitates the signals transmitted between neurons in biological brains [27]. Neurons in an ANN are divided into layers: 1 input layer, several hidden layers, and 1 output layer, where the number of neurons and hidden layers is not fixed. “Signals” transmitting is accomplished by weights and activation functions. As long as the initialized weights are updated by the self-learning process, the ANN can generate a “perfect” model. On behalf of the complex structure, an ANN can capture nonlinear associations and reveal potential interactions between variables. In our study, we structured an ANN with 5 hidden layers (neurons of each layer: 128-64-32-16-8), HeNormal weight initializer [28], ReLU activation function in the hidden layers, and sigmoid activation in the output layer [29]. These settings partially referred to previous studies [20,30] (Figure 1).

### Logistic Regression

Logistic regression (LR) estimates the probability of an event (outcome variable) taking place (success) based on a given data set of independent variables. In the LR equation, the outcome variable is transformed into log odds, the natural logarithm of the probability of success divided by the probability of failure. The independent variables are linearly structured by distributing coefficients to them. These coefficients are commonly estimated via maximum likelihood estimation to optimize the best fit of the log odds. The model is fixed once the optimal coefficients are found [31]. As a typical method that is widely used in epidemiological research, LR is introduced in this study to compare its performance with the novel ANN method.

### Data Processing

**Figure 2:**
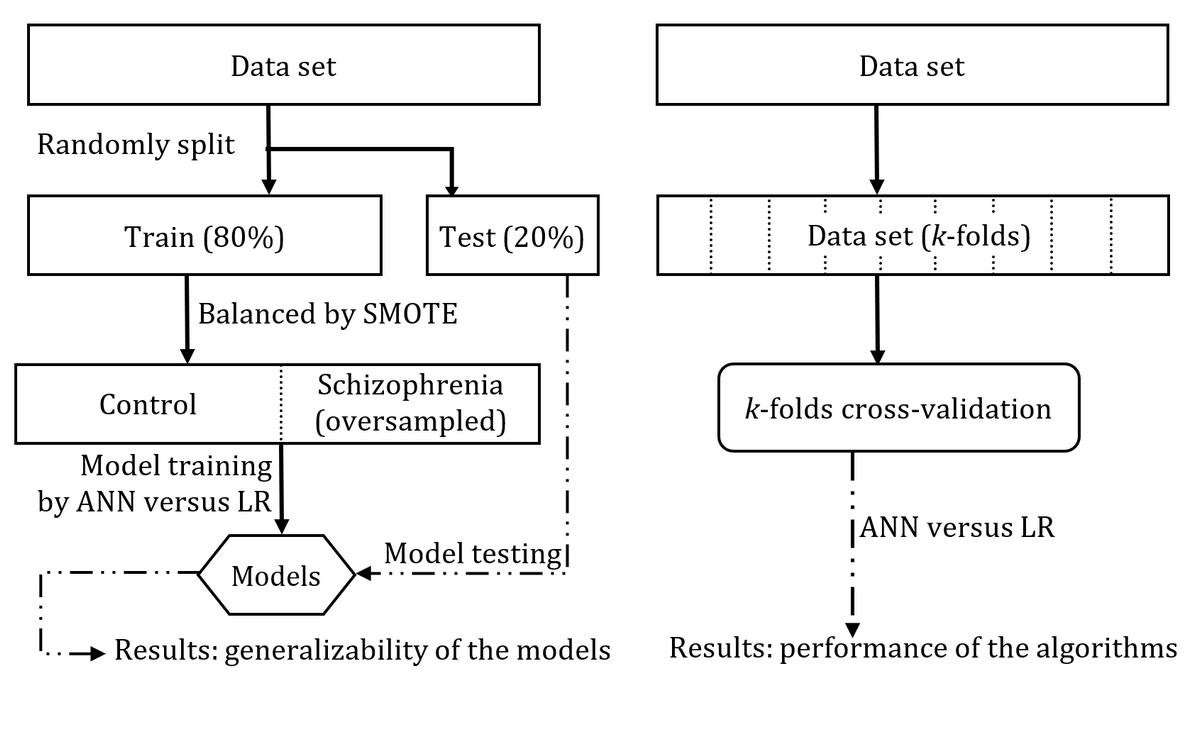
Flowchart of data processing, model training, and evaluation. ANN: artificial neural network; LR: logistic regression; SMOTE: synthetic minority oversampling technique.

The data were randomly split into a training data set and a test data set at an 80:20 ratio. Before applying the 2 selected algorithms (ANN and LR), the training data set was balanced based on the synthetic minority oversampling technique [32]. Models were trained using ANN and LR on the training data set separately, and the test data set was used to evaluate the 2 models (ANN and LR model). The area under the receiver operating characteristic curve (AUC) was applied to interpret the results because the outcome was binary. The 95% CI for the AUC was generated from 10,000 bootstraps of the training data set (Figure 2).

### Evaluation

The following thresholds were used to evaluate the performance in terms of AUC score: 0.5=no discrimination, 0.5-0.7=poor discrimination, 0.7-0.8=acceptable discrimination, 0.8-0.9=excellent discrimination, and >0.9=outstanding discrimination [33]. Two strategies were designed to evaluate the performance of the models and algorithms. To compare the generalizability of the trained LR and ANN models, their AUCs on the test data sets were compared. To compare the performance of LR and ANN algorithms, the AUCs were compared based on a 10-fold cross-validation. The differences in the AUCs were tested using the Delong method [34].

### Model Interpretation (Variable Importance)

To interpret the ANN model, we introduced a shuffle test for each variable to evaluate variable importance. Among all the *N* variables in the test data set, the *n*th variable is shuffled at random; this resampled test data set is applied to the ANN model, and the AUC obtained from the resampled test data set is compared with the AUC obtained from the original test data set. The difference between the 2 AUCs explains the importance of the variable. A higher difference indicates that the *n*th variable has relatively higher importance.

### Statistical Analyses

Statistical analyses were performed using Python (version 3.7; Python Software Foundation). The computational environment used was the Jupyter Notebook (Project Jupyter). Means and SDs are presented for continuous variables. Categorical variables are presented as proportions. Differences in means for continuous variables and categorical variables were tested using analysis of variance and chi-square test, respectively.

### Ethical Considerations

This study was approved by the Bioethics Review Committee of Fujita Health University (HM21-408). All procedures performed in this study were in accordance with the Ethical Guidelines for Medical and Health Research Involving Human Subjects enforced by the Ministry of Health, Labour and Welfare, Government of Japan, and the 1964 Helsinki Declaration and its later amendments.

## Results

Table 1 shows the characteristics of the normal and schizophrenia groups. Compared with the control group, participants with schizophrenia were more likely to be men. They had a significantly higher proportion of obesity, lower education levels, and lower household income. Participants with schizophrenia were more likely to have poor self-rated health status, depressive symptoms, perceived stress, and lower availability of social support. More details of these characteristics are provided in Table S1 in Multimedia Appendix 1.

**Table 1:** Characteristics of schizophrenia cases and healthy controls.

		Schizophrenia case (N=223)	Healthy control (N=1776)	*P* value^a^
Age (years), mean (SD)		46 (9.3)	44 (13.5)	.08
**Gender, n (%)**				
	Women	108 (48)	975 (55)	.07
BMI (≥25 kg/m^2^), n (%)		103 (46)	313 (18)	<.001
**Education, n (%)**				
	Junior or senior high school or lower	94 (42)	471 (27)	<.001
**Household income, n (%)**				
	<3 million Japanese yen^b^	108 (48)	359 (20)	<.001
**Self-rated health status, n (%)**				
	Bad	108 (48)	386 (19)	<.001
**Physical disease, n (%)**				
	≥1 disease	135 (61)	610 (31)	<.001
**Depressive symptoms, n (%)**				
	CES-D^c^ ≥8	156 (70)	467 (26)	<.001
Perceived stress (PSS-4^d^), median (IQR)		10 (8-12)	7 (6-8)	<.001^e^
Social support (ESSI^f^), median (IQR)		21 (15-28)	23 (17-28)	.007^e^

^a^Based on analysis of variance and chi-square test for continuous and categorical variables, respectively, except specified notes.

^b^Around US $20,000.

^c^CES-D: Center for Epidemiological Studies Depression.

^d^PSS-4: 4-item Perceived Stress Scale.

^e^*P* values by the Kruskal-Wallis test.

^f^ESSI: ENRICHD Social Support Instrument.

**Figure 3:**
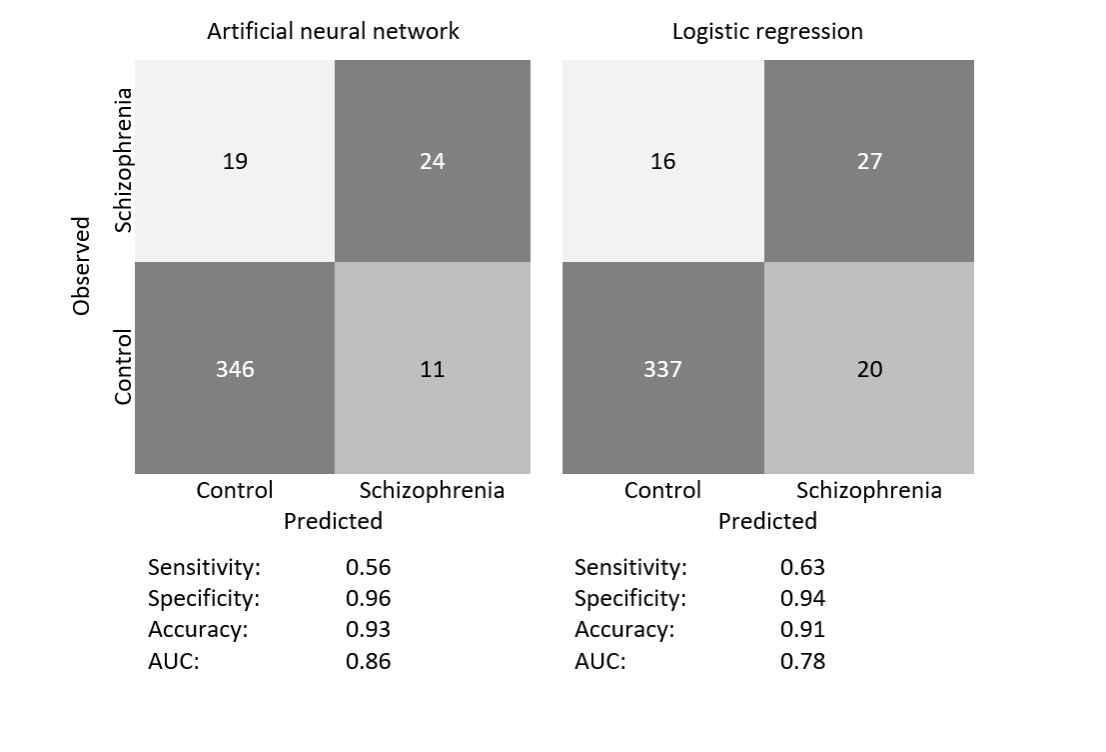
Confusion matrixes of artificial neural network and logistic regression models. AUC: area under the receiver operating characteristic curve.

Figure 3 illustrates the internal validity of the models trained using the ANN and LR. The model trained by ANN performed better than LR in terms of AUC (0.86 vs 0.78), accuracy (0.93 vs 0.91), and specificity (0.96 vs 0.94), whereas the model trained by LR performed better in terms of sensitivity (0.63 vs 0.56). Table 2 shows the algorithm performance comparing between ANN and LR by using bootstrapping and cross-validation. ANN performed better in terms of AUC (bootstrapping: 0.847 vs 0.773 and cross-validation: 0.81 vs 0.72), whereas LR performed better in terms of accuracy (0.894 vs 0.856).

**Table 2:** Comparison between artificial neural network and logistic regression by bootstrapping and cross-validation.

			Artificial neural network, mean (SD)		Logistic regression, mean (SD)		*P* value
**Results from 10,000 bootstrapping**							
	Accuracy	0.856 (0.18)		0.894 (0.01)		<.001	
	AUC^a^	0.847 (0.11)		0.773 (0.02)		<.001	
**Results from 10-fold cross-validation**							
	Accuracy	0.92 (0.04)		0.82 (0.28)		.27	
	AUC	0.81 (0.28)		0.72 (0.26)		.49	

^a^AUC: area under the receiver operating characteristic curve.

**Figure 4:**
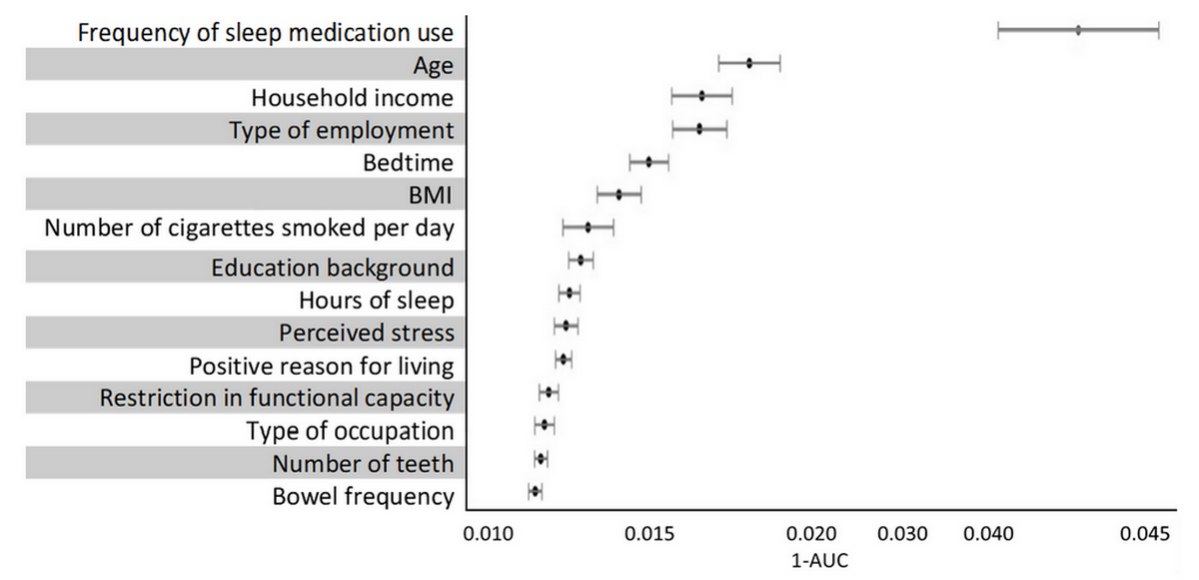
Feature importance (top 15) from artificial neural network model. AUC: area under the receiver operating characteristic curve.

Figure 4 shows the feature importance ranking estimated from the ANN model (only the top 15 items are displayed). The frequency of sleep medication use ranked first, illustrating the most important factor associated with schizophrenia. Age took second place. Household income and type of employment ranked third as they showed similar values. Bedtime, BMI, number of cigarettes smoked per day, and educational background followed, with a marked decrease in importance. Hours of sleep, perceived stress, positive reason for living (aka, *ikigai*, a Japanese term), restriction in functional capacity, type of occupation, number of teeth, and bowel frequency ranked 9th to 15th; however, their importance was not as high as those in the above places.

## Discussion

### Principal Findings

In this study, we developed an ANN model for classifying schizophrenia cases with high internal validity, which achieved an excellent AUC of 0.86. This ANN model also achieved a specificity of 0.96, which implies that it has a good ability to designate an individual without schizophrenia as negative, and a sensitivity of 0.56, which represents the model’s limitation in designating an individual with schizophrenia as positive. Our study demonstrated that the ANN has the potential for applying to estimate the prevalence of schizophrenia in large-scale epidemiological studies.

To our knowledge, this is the first study that uses a machine learning technique (ie, ANN) to classify schizophrenia cases from web-based survey data in the Japanese population. A novel machine learning approach has been reported for the detection of schizophrenia from social media content, which achieved an accuracy of 96% [16]. In addition, machine learning techniques might provide an opportunity to improve diagnostic certainty [35] and explain mental disorders in complex states [36]. On the other hand, ANN algorithms have proven their advantages in disease prediction using large-scale survey data. For predicting type 2 diabetes, models developed by ANN have achieved an AUC of 0.86, which is the highest compared to other algorithms such as random forest and support vector machine [21]. Another study reported that models developed by ANN for predicting hypertension achieved an AUC of 0.78; however, there was no significant advantage compared with the classic method [20].

In comparison of the algorithm performance, ANN performed better than LR. Several reasons might be considered: (1) ANN is more competent for approximate relations that do not follow the linearized assumption owing to its structure [37]. In generalized linear models (eg, LR), the relation between the response variable and the feature variables is applied to a linear equation; therefore, linear models face difficulty in analyzing nonlinear combinations. As the number of feature variables in a linear model increases, multicollinearity [38] and overfitting may occur easily [39]. (2) The ANN is more effective for analyzing interactions among more than 2 variables. In linear models, interaction terms (usually products between 2 variables) are added. However, interaction features are often selected based on rules of thumb. Additionally, multicollinearity should be cautiously considered as the number of interaction terms increases [38]. While in an ANN, the variable relationships are assumed to be extremely high-dimensional and complex [40]. After inputting all the variables, each hidden neuron takes, as input, all nodes from the previous layer and creates a high-order interaction between these nodes [41]. Nevertheless, the complex structure of an ANN prevents the model from being easily visualized and understood.

In terms of the AUC value, ANN outperformed LR both in algorithm and model comparisons. However, in this study, the ANN model exhibited better specificity but lower sensitivity compared to LR as the cutoff threshold was set to the default 0.5. The model's performance metrics indicate a trade-off between sensitivity and specificity. This trade-off could impact the model's ability to correctly classify schizophrenia cases and noncases, which has clinical implications. Further experiments are necessary, involving the adjustment of the cutoff threshold [42] to determine a suitable balance point in practical scenarios.

This study suggested the possibility of classifying schizophrenia cases among general population. We expected to estimate schizophrenia cases among those who are not seeking psychiatric care, typically, whose psychiatric syndromes are unknown. Hence, no clinical assessment or diagnostic criteria were involved in the feature variables. As we ranked the feature importance, sleep problems were the most important factor associated with schizophrenia in terms of sleep medication use, bedtime, and sleep duration. The importance of factors such as age, socioeconomic background, BMI, physical activity, smoking, depression, and oral health followed. These factors have also been reported to be strongly associated with schizophrenia [8,43-50]. The advantage of our study was that we ranked those previously reported factors according to their “priority” for classifying schizophrenia, which may provide potential evidence for screening and early detection of schizophrenia when using massive data.

In this study, no variable selection technique was preselected because we hope to mine as much potentially useful information as possible when training the models. Previous studies have reported that fully input variables might lead to unstable estimates in linear models such as LR [51]. Some methodologists have suggested that statistical significance–based variable selection techniques are mechanical and, as such, have limitations [51,52]. We conducted an additional experiment for the sake of “fairness”; the LR model was trained using only the top 15 most important features reported by the ANN model. The results obtained from this partial-featured LR model did not improve compared with the all-feature LR model (sensitivity: 0.69, specificity: 0.87, accuracy: 0.91, and AUC: 0.78). The machine learning approach might be used for feature selection to compensate for the limitations of classical epidemiology studies.

### Limitations

This study has several limitations. First, participants with mental disorders other than schizophrenia were not included. Hence, our model is not suitable for distinguishing schizophrenia from other types of mental disorders. Second, although the data in this study were obtained from a large Japanese internet research pooled panel, we should cautiously explain the representativeness for the entire Japanese population. This could introduce sampling bias, potentially excluding individuals who are less likely to participate in web-based surveys, such as those with limited internet access or severe mental health conditions. In addition, the self-reported diagnoses of schizophrenia might not be as reliable as clinically confirmed diagnoses, as individuals might misinterpret symptoms or misunderstand their condition. There might also be issues related to stigma or disclosure bias, where individuals might be hesitant to disclose mental health diagnoses. Third, the important variables determined by our model cannot be arbitrarily used as a criterion for identifying schizophrenia. For example, sleep medication use is often observed in patients with depression, although our model determined that it was most associated with schizophrenia. In future studies, we plan to include samples with various types of mental disorders and construct a model that can classify multiple mental disorders. Additionally, Clinical assessments and diagnostic criteria used by health care professionals were not included in the analysis. In future studies, we can introduce essential information to enhance the model construction. Fourth, all variables (feature variables and response variables) were self-reported at the same time. The answers were possibly biased because the participants might have been reluctant to answer some sensitive questions truthfully. The existence of schizophrenia might be different from the actual situation because the history of schizophrenia was reported by the participants themselves. Patients who did not use the internet and those who had difficulty completing the web-based survey due to their illness were not included in this study. These issues may have affected the accuracy and generalizability of the model. Fifth, the findings of this study were derived from a cross-sectional design; therefore, it is difficult to explain any causal or temporal associations. Sixth, because of the “black-box” design of the ANN model, it is difficult to interpret how variables and variable interactions contribute to the classification of schizophrenia. Further research is necessary to focus on model visualization and interpretation. Finally, the ideal model should be dynamic (ie, can be updated to adopt the latest data structure) [53]; hence, we need to input more large-scale data to improve the current model and to assess the model performance by external validation.

### Conclusions

In this study, an ANN model was constructed to classify schizophrenia cases using web-based survey data. The model achieved a high internal validity. ANN performed better compared to the classical statistic method. These findings are expected to provide evidence for estimating the prevalence of schizophrenia in the Japanese population and informing future epidemiological studies.

## Appendix

Multimedia Appendix 1 Data source and variable definition.

## Abbreviations

ANN: artificial neural network
AUC: area under the receiver operating characteristic curve
LR: logistic regression

## Data Availability

The data sets analyzed during this study are available from the corresponding author on reasonable request.
