# Supplementary material for: Classifying Schizophrenia Cases by Artificial Neural Network Using Japanese Web-Based Survey Data: Case-Control Study
Source: JMIR Form Res. 2023 Nov 15;7:e50193. doi: 10.2196/50193 (PMC10687680; doi:10.2196/50193)
Supplement: Multimedia Appendix 1 [file formative_v7i1e50193_app1.docx]

**Multimedia Appendix 1.** Data source and variable definitions.

**Data source**

Data were collected from an internet research agency’s pooled panels (Rakuten Insight, Inc., incorporated approximately 2.3 million panelists by 2022) [1]. A prevalence case-control study was conducted using that panels. Subjects’ age was limited from 20- to 75-year-old.

For involving subjects who currently have schizophrenia, 5,584 individuals who self-reported schizophrenia were sampled in the Rakuten Insight disease panel. 3,256 respondents answered the following four questions in advance of the survey: (1) Are you currently suffering from schizophrenia only, schizophrenia and migraine, schizophrenia and a sleep disorder, or schizophrenia, migraine, and a sleep disorder? (2) Have you experienced auditory hallucinations lasting more than one month? (3) Have you never used stimulants or other illegal drugs and never been an alcoholic? (4) Have you experienced the first auditory hallucination lasting more than one month at less than 60 years of age? Those who answered “yes” to all four questions were considered to currently have schizophrenia. Therefore, 223 respondents were involved in the survey.

For involving subjects who do not currently have schizophrenia, 28,000 participants in the Japan COVID-19 and Society Internet Survey (JASTIS, which was also conducted using the Rakuten Insight Panel) [2-4] were sampled. 6,656 respondents answered the following four questions in advance of the survey: (1) Are you currently suffering from mental illness? (2) Have you had mental illness in the past? (3) Have you experienced auditory hallucinations? (4) Have you ever used stimulants or other illegal drugs, been an alcoholic, or received psychiatric treatment? Those who answered “no” to all four questions were considered not to have schizophrenia. Therefore, 1,776 respondents were involved in the survey.

Finally, 223 Subjects aged 20-75 years who currently have schizophrenia and 1776 health controls were included in this study.

**Feature variables**

Feature variables were formatted from a self-administered questionnaire assessed 1) demographic, health-related backgrounds, and physical comorbidities, 2) psychiatric comorbidities, and 3) social comorbidities.

**1) Demographic, health-related backgrounds, and physical comorbidities**

Demographic, health-related backgrounds, and physical comorbidities include **age**, **body mass index** (BMI), **smoking status** [current, past, never], **number of cigarettes per day**, **alcohol drinking frequency** [never, past, current: <1 day in one month; 1-3 days in one month; 1-2 days in one week; 3-4 days in one week; 5-6 days in one week; everyday.], **sports** [frequency: < once in one month; 1-3 times in one month; 1-2 times in one week; 3-4 times in one week; almost every day. Intensity: each time < 30 minutes; 30-59 minutes; 1-2 hours; 2-3 hours; 3-4 hours; >4 hours], **eating behaviors** [tendency to overeat: yes/no; eating speed: very fast, fast, normal, slow, very slow; having breakfast, eating out, and eating instant foods: < once in one month; 1-3 times in one month; 1-2 times in one week; 3-4 times in one week; almost every day], **bowel movement** [frequency: <3times per week, 3-4 times per week, 5-6 times per week, once a day, > once a day], **Stool** [soft, normal, hard, recurrent diarrhea and constipation], **restriction in functional capacity** [5], **self-rated health status** (SRHS) [6], and **physical comorbidities** [overweight, cancer, cardiovascular disease, heart failure, hypertension, diabetes, dyslipidemia, gout, sleep apnea syndrome, and fracture: have been treated or not].

**Restriction in functional capacity**

To evaluate functional capacity restrictions, we used the Scale of Independence in Daily Living for the Disabled Elderly published by the Ministry of Health, Labour and Welfare, Japan [5]. The term ”restrictions in functional capacity” refers to a multidimensional concept that involves sensory loss, impaired mobility, vascular disorders, gait impairments, problems with daily living (ADLs), and changes in body systems. Participants self-assessed restrictions by choosing one of the following options: (1) “I have no physical disabilities,” (2) “I go out alone, using transportation,” (3) “I can only go out alone in my neighborhood,” (4) “I go out with help and live mostly out of bed during the day,” (5) “I can go out with help, but I go out infrequently, and I spend most of the daytime sleeping on and off in bed,” (6) “I can ride in a wheelchair by myself and eat and toilet away from the bed,” (7) “I can ride in a wheelchair with assistance.,” (8) “I can roll over in bed,” and (9) “I cannot roll over in bed.”

**Self-rated health status (SRHS)**

SRHS is a self-reported measure of health status that incorporates a person’s biological, mental, social, and functional aspects, including individual and cultural beliefs and health behaviors. It is a strong predictor of all-cause mortality in general populations [6]. Participants responded to the question “What do you think of your general health status during the previous month?” by choosing one of the following options: “great,” “pretty good,” “good,” “not so good,” and “bad.”

**2) Psychiatric comorbidities**

Psychiatric comorbidities include **frequency of sleep medication use** [never, < 1 day per week, 1-2 days per week, 3-4 days per week, 5-6 days per week, every day], **bedtime** [before 7:00pm, around 8:00pm, around 9:00pm, around 10:00pm, around 11:00pm, around 0:00am, around 1:00am, around 2:00am, around 3:00am, after 4:00am, not fixed], **sleep hours** [≤5 hours, 6 hours, 7 hours, 8 hours, 9 hours, ≥10 hours], **hypnagogic disorder frequency** [almost never, < 1 time per week, 1-2 times per week, 3-4 times per week, 5-6 times per week, almost every day], **deep sleep disorder frequency** (waking up tired) [almost never, < 1 time per week, 1-2 times per week, 3-4 times per week, 5-6 times per week, almost every day], **middle wakening, or early wakening frequency** [almost never, < 1 time per week, 1-2 times per week, 3-4 times per week, 5-6 times per week, almost every day], **depressive symptoms** [7,8], **perceived stress** [9], ***ikigai*** (a Japanese term which means positive reason for living), **happiness**, and **internet use hours per week**.

**Depressive symptoms (CES-D)**

We used a modified 11-item Center for Epidemiological Studies Depression (CES-D) Scale in this study [7,8]. The existence of depressive symptoms was defined as a score of 8 or higher.

**Perceived stress (PSS-4)**

We assessed perceived stress with a 4-item Perceived Stress Scale (PSS-4) [9]. Scores are on a 16-point scale, with higher total scores indicating more severe perceived stress. Perceived stress was defined as being present when the score was higher than 7, the median of the PSS-4 scores for non-schizophrenic participants.

**Ikigai**

The Japanese term “Ikigai” is a positive reason for living. Participants were asked, “Do you have any positive reasons to live?” and answered the question from four options: “very much so,” “yes,” “no,” or “not at all.” Participants who answered “no” or “not at all” were defined as absent of ikigai.

**Happiness**

Participants were asked, “How happy do you feel about your life?” and answered the question from four options: “very happy,” “happy,” “neither happy nor unhappy,” and “unhappy.” Participants who answered “neither happy nor unhappy” or “unhappy” were defined as absence of happiness.

**3) Social comorbidities**

Social comorbidities include **taking regular health checkups** [yes/no], **educational background** [junior/senior high school, university, junior college, or vocational school], **type of occupation** [unemployed, homemaker, white-collar workers, or blue-collar workers], **type of employment** [regular, irregular, or self-employed/business people], **household income** [< 3, 3–6, 6–9, 9-12-15, ≥ 15 million Japanese Yen], **marital status** [unmarried, married, divorced, widowed, others], **family structure** [living alone, living with parents, living with spouse, living with children, and living with other people; how many people living with], **social support** [10,11], and **social capital** (cognitive and structural dimensions) [12,13].

**Social support**

We assessed social support with the ENRICHD Social Support Instrument (ESSI) [10,11]. A higher total score indicates higher availability of social support.

**Social capital**

To assess social capital, we partly referred to the Integrated Questionnaire for the Measurement of Social Capital (SC-IQ) [12]. It is common practice in social capital research to distinguish between structural and cognitive dimensions [13].

We focused on cognitive social capital using the following SC-IQ items: (1) “Can most people be trusted?”; (2) “Does one have to be alert or is someone likely to take advantage of you?”; and (3) “Are most people willing to help if you need it?” Responses were selected from four categories: “strongly disagree,” “disagree,” “agree,” and “strongly agree.” For the three questions, cognitive social capital was defined as high when there were two or more responses of “agree” or “strongly agree” to question (1), “disagree” or “strongly disagree” to question (2), and “agree” or “strongly agree” to question (3).

We also focused on structural social capital using the following SC-IQ items: “How often do you participate in community organizations, self-help groups, charities, volunteer groups, or religious gatherings?” The response was selected from four categories: “not at all/ very seldom,” “sometimes,” “less than once a week,” and “more than once a week.” Structural social capital was defined as high when the response was “more than once a week.”

**References**

1． Rakuten Insight, Inc. <https://insight.rakuten.co.jp/en/aboutus.html>. Accessed Oct 17 2023.

2. Kusama T, Kiuchi S, Takeuchi K, et al. Information Usage and Compliance with Preventive Behaviors for COVID-19: A Longitudinal Study with Data from the JACSIS 2020/JASTIS 2021. *Healthcare*. 2022;10(3):521. doi:10.3390/healthcare10030521

3. Wakabayashi M, Sugiyama Y, Takada M, Kinjo A, Iso H, Tabuchi T. Loneliness and Increased Hazardous Alcohol Use: Data from a Nationwide Internet Survey with 1-Year Follow-Up. *Int J Environ Res Public Health*. 2022;19(19):12086. doi:10.3390/ijerph191912086

4. Sasaki R, Ota A, Yatsuya H, Tabuchi T. Gender Difference in Fear and Anxiety about and Perceived Susceptibility to COVID-19 in the Third Wave of Pandemic among the Japanese General Population: A Nationwide Web-Based Cross-Sectional Survey. *Int. J. Environ. Res. Public Health*. 2022;19(23):16239. doi:10.3390/ijerph192316239

5. The Ministry of Health, Labour and Welfare. The Standards for the Degree of Independent Living for Elderly And Disabled People. 1991. <https://www.mhlw.go.jp/file/06-Seisakujouhou-12300000-Roukenkyoku/0000077382.pdf>. Accessed Oct 17 2023 (in Japanese).

6. DeSalvo KB, Bloser N, Reynolds K, He J, Muntner P. Mortality prediction with a single general self-rated health question. *J Gen Intern Med*. 2006;21(3):267-275. doi:10.1111/j.1525-1497.2005.00291.x

7. Radloff LS. The CES-D scale. *Applied Psychological Measurement*. 1977;1(3):385-401. doi:10.1177/014662167700100306

8. Kohout FJ, Berkman LF, Evans DA, Cornoni‐Huntley J. Two shorter forms of the CES-D Depression Symptoms index. *J Aging Health*. 1993;5(2):179-193. doi:10.1177/089826439300500202

9. Warttig SL, Forshaw M, South J, White A. New, normative, English-sample data for the Short Form Perceived Stress Scale (PSS-4). *J Health Psychol*. 2013;18(12):1617-1628. doi:10.1177/1359105313508346

10. The ENRICHD Investigators. Enhancing recovery in coronary heart disease (ENRICHD) study intervention: rationale and design. *Psychosom Med*. 2001; 63: 747–755. PMID: 11573023.

11. Enhancing Recovery in Coronary Heart Disease Patients (ENRICHD): Study design and methods. *American Heart Journal*. 2000;139(1):1-9. doi:10.1016/s0002-8703(00)90301-6

12. Grootaert G, Narayan D, Jones VN, Woolcock M. Measuring Social Capital : an Integrated Questionnaire. *World Bank Publications*. January 2004. https://ideas.repec.org/b/wbk/wbpubs/15033.html.

13. Murayama H, Fujiwara Y, Kawachi I. Social Capital and Health: A review of Prospective Multilevel studies. *J Epidemiol*. 2012;22(3):179-187. doi:10.2188/jea.je20110128

**Table S1.** Characteristics of schizophrenia cases and healthy controls^a^.

|  | Schizophrenia case  N=223 | Healthy control  N= 1776 | *p*-value ^b^ |
| --- | --- | --- | --- |
| Age years, mean±SD | 46±9.3 | 44±13.5 | 0.08 |
| Sex  women | 108 (48%) | 975 (55%) | 0.07 |
| BMI  ≥ 25 kg/m^2^ | 103 (46%) | 313 (18%) | <0.001 |
| Smoking status  Current | 53 (24%) | 304 (17%) | 0.02 |
| Alcohol drinking  Current | 48 (22%) | 676 (38%) | <0.01 |
| Sports  ≥ 1 times per week | 91 (41%) | 795 (45%) | 0.26 |
| Tendency to overeat | 140 (63%) | 975 (55%) | 0.03 |
| Eating out  ≥ 1 times per week | 69 (31%) | 734 (41%) | <0.01 |
| Eating instant food  ≥ 5 times per week | 15 (6.7%) | 64 (3.6%) | 0.04 |
| Bowel motion  < 3 times per week | 21 (9.4%) | 165 (9.3%) | 0.96 |
| Stool  Soft and normal | 186 (83%) | 1532 (86%) | 0.26 |
| Sleep hours  ≥ 10 hours per day | 23 (10%) | 17 (1.0%) | <0.01 |
| Restrictions in functional capacity | 89 (40%) | 104 (5.9) | <0.001 |
| Self-rated health status  Bad | 108 (48%) | 386 (19%) | <0.001 |
| Physical disease  ≥ 1 disease | 135 (61%) | 610 (31%) | <0.001 |
| Regular health checkups  Yes | 95 (43%) | 1063 (60%) | <0.001 |
| Education  Junior/senior high school or lower | 94 (42%) | 471 (27%) | <0.001 |
| Type of occupation  Unemployed | 94 (42%) | 185 (10%) | <0.001 |
| Type of employment  Irregular | 64 (66%) | 377 (28%) | <0.001 |
| Household income  <3 million Japanese yen (around 20,000 USD) | 108 (48%) | 359 (20%) | <0.001 |
| Marital status  Married | 62 (28%) | 982 (55%) | <0.001 |
| Family structure  Living alone | 33 (15%) | 368 (21%) | 0.04 |
| Social support (ESSI) ^c^ | 21 (15,28) | 23 (17,28) | <0.01 |
| Depressive symptoms  CES-D >=8 | 156 (70%) | 467 (26%) | <0.001 |
| Hypnagogic disorder  < 1 time per week | 106 (48%) | 1241 (70%) | <0.001 |
| Deep sleep disorder  < 1 time per week | 71 (32%) | 885 (50%) | <0.001 |
| Middle or early wakening  < 1 time per week | 101 (45%) | 1261 (71%) | <0.001 |
| Perceived stress (PSS-4) ^c^ | 10 (8,12) | 7 (6,8) | <0.001 |
| Social support (ESSI) ^c^ | 21 (15,28) | 23 (17,28) | <0.01 |
| Ikigai  Present | 97 (43%) | 1128 (64%) | <0.001 |
| Happiness  Present | 98 (44%) | 1191 (67%) | <0.001 |
| Internet use hours per week ^c^ | 16 (7, 35) | 14 (6, 22) | <0.01 |
| Social capital |  |  |  |
| Less cognitive social capital | 128 (53%) | 804 (45%) | <0.001 |
| Less structural social capital | 189 (85%) | 1536 (86%) | 0.54 |

Abbreviations: CSE-D, Center for Epidemiological Studies Depression; ESSI, ENRICHD Social Support Instrument; PSS-4, 4-item Perceived Stress Scale; SD, standard deviation.

^a^Data are presented as means ± standard deviations or n (percentages) for continuous or categorical variables, respectively.

^b^Based on analysis of variance and Chi-2 test for continuous and categorical variables, respectively, except specified notes.

^c^Data are presented as medians (inter quartile ranges) and *p*-values by Kruskal-Wallis test.
